# Structures of *S*-(pyridin-2-yl) 4-nitro­benzo­thio­ate, *S*-(pyridin-2-yl) 4-methyl­benzo­thio­ate and *S*-(pyridin-2-yl) 4-meth­oxy­benzo­thio­ate: building blocks for low-symmetry multifunctional tetra­pyrroles

**DOI:** 10.1107/S2056989023001056

**Published:** 2023-02-09

**Authors:** Harry C. Sample, Brendan Twamley, Mathias O. Senge

**Affiliations:** a School of Chemistry, Chair of Organic Chemistry, Trinity Biomedical Sciences Institute, Trinity College Dublin, 152-160 Pearse St, D02 R590, Dublin, Ireland; b School of Chemistry, Trinity College Dublin, College Green, Dublin 2, Ireland; Universität Greifswald, Germany

**Keywords:** crystal structure, benzo­thio­ate derivatives, hydrogen bonding, π–π-stacking

## Abstract

The crystal structures of three *S*-(pyridin-2-yl) benzo­thio­esters with varying *para*-phenyl substituents are presented, namely, *S*-(pyridin-2-yl) 4-nitro­benzo­thio­ate (**1**, C_12_H_8_N_2_O_3_S), *S*-(pyridin-2-yl) 4-methyl­benzo­thio­ate (**2**, C_13_H_11_NO_2_S) and *S*-(pyridin-2-yl) 4-meth­oxy­benzo­thio­ate (**3**, C_13_H_11_NO_2_S). This class of compounds are used in the mono-acyl­ation of pyrrolic species to yield multifunctional tetra­pyrroles. The structures presented herein are the first of their compound class. The dominant inter­actions present in this series are π–π stacking and C—H⋯O inter­actions, and as the *para*-phenyl motif changes from electron withdrawing (NO_2_, **1**) to electron donating (OCH_3_, **3**), changes are observed in the inter­actions present in the crystal packing, from predominant π–π stacking in **1** to exclusively C—H⋯O/N inter­actions (C_ar­yl_—H⋯O_carbon­yl_, C—H⋯O_meth­oxy_ and C_ar­yl_—H⋯N_pyridine_) in **3**.

## Abstract 2

The structures of three *S*-(Pyridn-2-yl) benzo­thio­esters are presented with varying *para*-phenyl motifs (NO_2_ in **1**, CH_3_ in **2**, and OCH_3_ in **3**). These structures presented are the first in their class. Distinct changes are observed in the inter­action types present in the crystal lattice as a direct result of the electronic influence of the *para*-phenyl motif.

## 1. Chemical context

**Figure 1:**
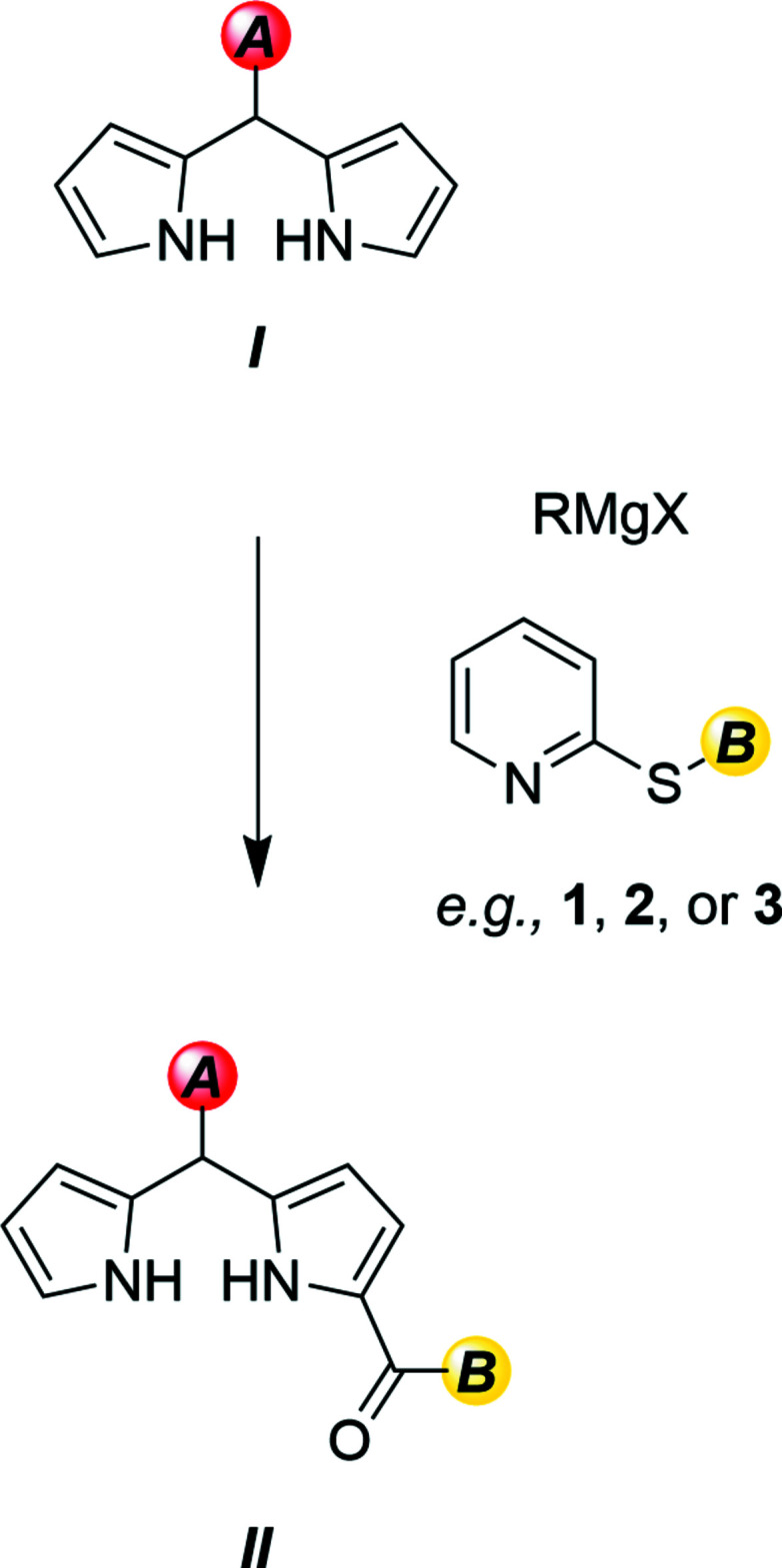
Transformation of simple *meso*-substituted dipyrro­methanes (***I***) to monoacyl-dipyrro­methanes (***II***) through the use of *S*-(pyridin-2-yl) thio­esters. *A*, *B* = aryl, *R* = Et, *i*Pr, *X* = Br, Cl.

In the continual search of evermore functional tetra­pyrroles, the tedious separation of multiple regioisomeric porphyrins from mixed Adler–Longo (Adler *et al.*, 1967 ▸) or Lindsey-style syntheses (Lindsey *et al.*, 1986 ▸) no longer suits the desires of the few in this research field. Instead, multiple elegant yet simple routes have been developed for the functionalization of the porphyrin core (Hiroto *et al.*, 2017 ▸; Sample *et al.*, 2021 ▸), as well as from the modification of pyrrolic precursors (Lindsey, 2010 ▸). One route of note is *via* the mono­acyl­ation of *meso*-substituted dipyrro­methanes (***I***, Fig. 1 ▸). Initially reported with the use of acyl chlorides by Lindsey and coworkers (Lee *et al.*, 1995 ▸), the procedure also yields the di­acyl­ated products in substantial yield. The same group reported the selective mono­acyl­ation of *meso*-aryl dipyrro­methanes through the use of *S*-(pyridin-2-yl) benzo­thio­esters (Rao *et al.*, 2000 ▸).

*S*-(Pyridin-2-yl)benzo­thio­esters were first synthesized for the determination of ionization constants for heterocyclic substances (Albert & Barlin, 1959 ▸). This methodology was later elaborated upon to generate a wide variety of alkyl, aryl and heteroaryl ketones (Araki *et al.*, 1974 ▸). These compounds were also utilized to generate 2-keto­pyrroles (Nicolau *et al.*, 1981 ▸). Their versatility was recently highlighted (Lee, 2020 ▸). The developments that have led to this point now enable the generation of diverse substitution patterns for both porphyrins (Rao *et al.*, 2000 ▸; Senge, 2011 ▸) and chlorins (Laakso *et al.*, 2012 ▸ *;* Ra *et al.*, 2015 ▸; Senge *et al.*, 2021 ▸). 
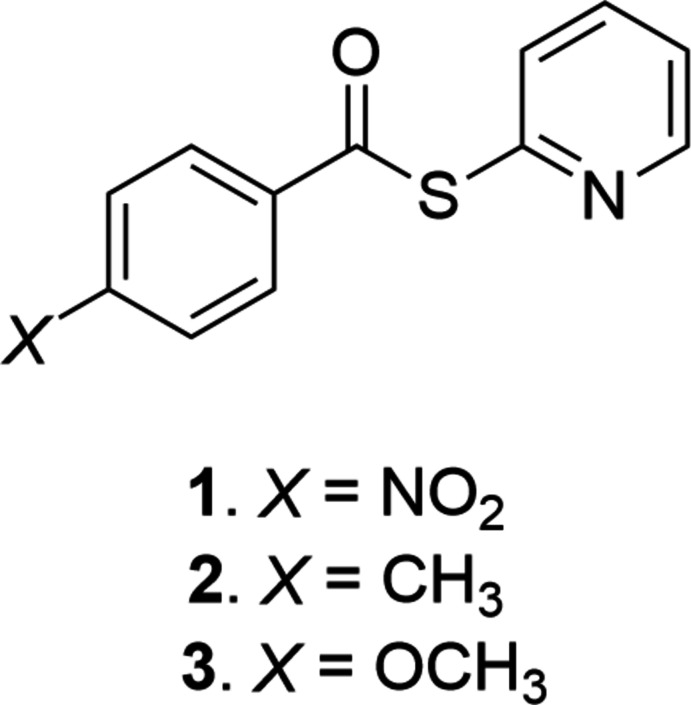


## 2. Structural commentary

**Figure 2:**
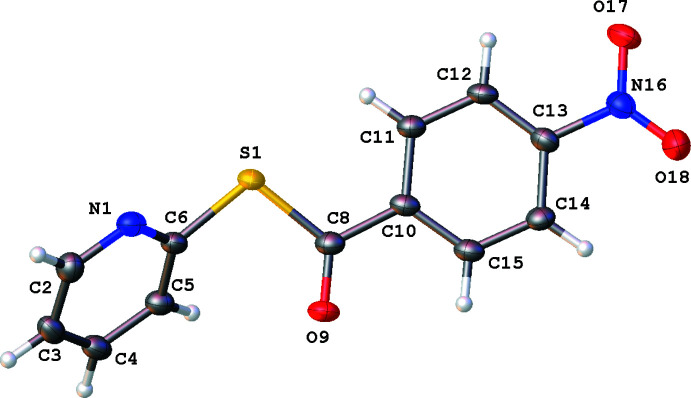
Mol­ecular structure of **1**. Anisotropic displacement ellipsoids are drawn at the 50% probability level. Generated using *OLEX2*.

The single-crystal XRD structures of title compounds **1**, **2** and **3** (Figs. 2 ▸–4 ▸ ▸), all present asymmetric units consisting of one mol­ecule of compound and no solvate. Compound **1** was found to crystallize in the ortho­rhom­bic system (*Pna*2_1_, *Z* = 4), compound **2** was found to crystallize in the triclinic system (*P* 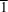 , *Z* = 2) and compound **3** was found to crystallize in the monoclinic system (*P*2_1_/*c*, *Z* = 4). Each mol­ecular structure shows an *S*-(pyridin-2-yl) benzo­thio­ate where the *para*-phenyl motif is modified, from NO_2_ in **1**, CH_3_ in **2**, and OCH_3_ in **3**. All of the groups utilized herein are found extensively in the field of tetra­pyrroles.

In all structures **1**–**3**, the substituted phenyl moieties are all essentially planar with the pyridine ring twisted relative to this plane. This is seen in the plane normal to plane normal angle and the torsion angle described by C8—S1—C6—N1. The twist of the methane­thio­ate moiety to the phenyl ring also describes the change in the angle of the rings to each other. These values are shown in Table 1 ▸.

**Figure 3:**
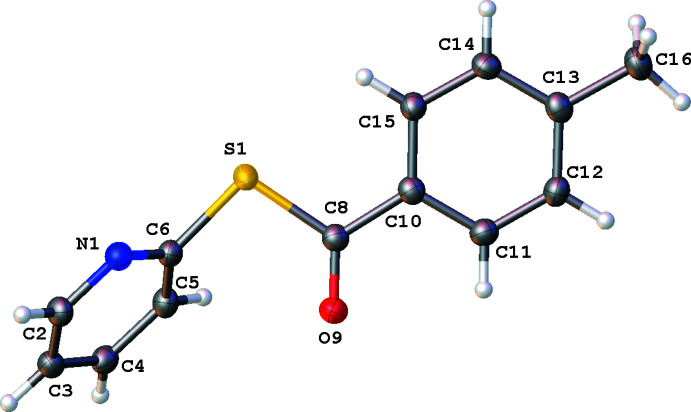
Mol­ecular structure of **2**. Anisotropic displacement ellipsoids are drawn at the 50% probability level. Generated using *OLEX2*.

**Figure 4:**
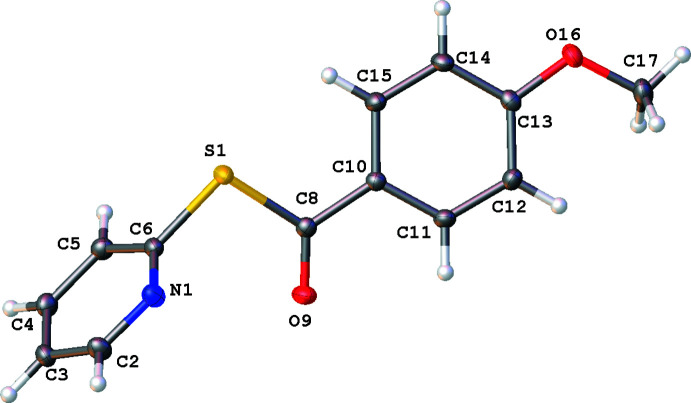
Mol­ecular structure of **3**. Anisotropic displacement ellipsoids are drawn at the 50% probability level. Generated using *OLEX2*.

In compound **1** (Fig. 2 ▸), the angle between the *para*-nitro­benzaldehyde moiety, C8–O18, and the pyridine ring is similar to the angle between the benzaldehyde moiety, C8–C16 and the pyridine ring in compound **2** (Fig. 3 ▸). The phenyl plane–pyridine plane angle and C8–S1–C6–N1 torsion angle in **3** (Fig. 4 ▸) are very different to those of both **1** and **2.**

**Figure 5:**
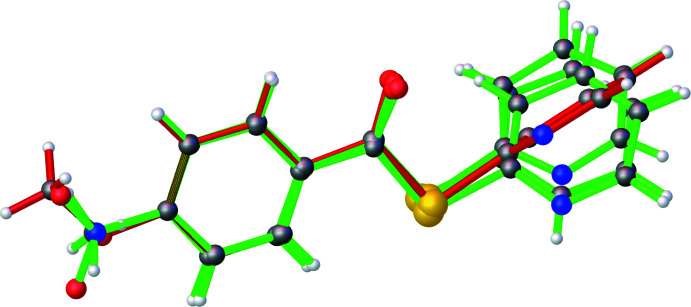
Overlay of **1**–**3** and CEFMOR showing the orientation of the pyridine ring in **3** (red) relative to the other structures. Generated using *OLEX2*.

All three benzo­thio­esters are similar to the previously published unsubstituted *S*-phenyl benzo­thio­ate (refcode: CEFMOR; Belay *et al.*, 2012 ▸). An overlay of **1**–**3** with CEFMOR is provided as Fig. 5 ▸. The bond distances are within normal ranges (Groom *et al.*, 2016 ▸).

## 3. Supra­molecular features

Of the varying *para*-phenyl motifs presented across the series, the NO_2_ group in **1** is the most electron withdrawing, according to its tabulated Hammett constant (σ_p_ = 0.78; McDaniel & Brown, 1958 ▸) but also observed by the differing shifts in the resonances presented for the *para-*substituted phenyl ring, with extensive deshielding of the respective protons (Figs. S1, S4 in the supporting information). Furthermore, considering the respective previously determined Hammett constants, it is observed that the most electron donating is the OCH_3_ group in **3** (σ_p_ = −0.27), with **2** (CH_3_) lying somewhere in between (σ_p_ = −0.17) (McDaniel & Brown, 1958 ▸); again, this is reflected in the ^1^H NMR spectra.

**Figure 6:**
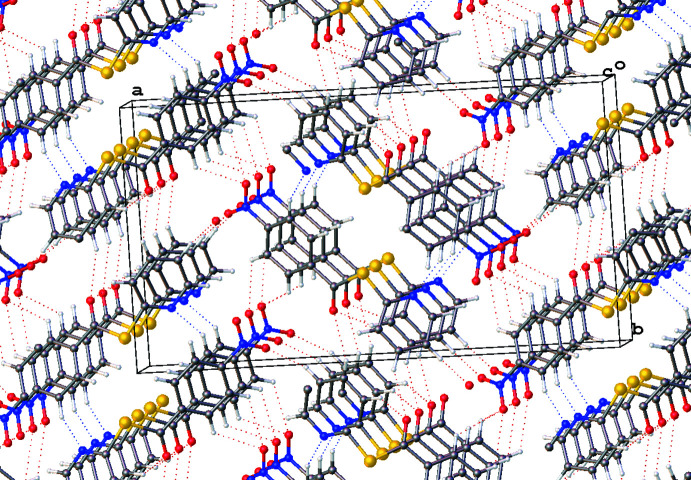
Excerpt of the packing structure of **1** viewed in the direction of the π-stack normal. Generated using *OLEX2*.

Compound **1** presents C—H⋯O inter­actions (Table 2 ▸, Fig. 6 ▸) to the carbonyl O9 *via* C4-H and C5-H donors [*D*⋯*A* = 3.283 (4) and 3.371 (5) Å]. The pyridine N1 is also an acceptor to the phenyl C12-H [*D*⋯*A* = 3.315 (5) Å]. The nitro group is a dual acceptor with inter­actions between O18 and one pyridyl C3-H [*D*⋯*A* = 3.396 (5) Å] and also a bifurcated inter­action between O17 and phenyl C14-H and C15-H [*D*⋯*A* = 3.359 (4) and 3.312 (5) Å, respectively].

**Figure 7:**
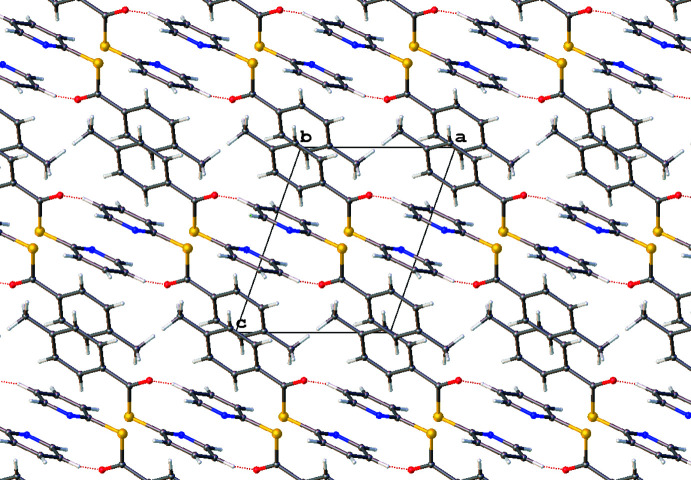
Hydrogen bonding, represented by dashed lines, shown in the packing structure of **2.** Viewed in the normal to the *b* axis. The pairs of offset π-π phenyl rings are also evident. Generated using *OLEX2*.

Compound **2** presents C—H⋯N-paired dimers between the H^6^-pyridyl protons C2-H2 and N1 [*D*⋯*A* = 3.355 (2) Å; Table 3 ▸, Fig. 7 ▸]. The carbonyl is involved in a bifurcated inter­action C3-H/C4-H⋯O9 [*D*⋯*A* = 3.278 (2) and 3.316 (2) Å, respectively] and a C16-H⋯O9 inter­action [*D*⋯*A* = 3.460 (2) Å].

**Figure 8:**
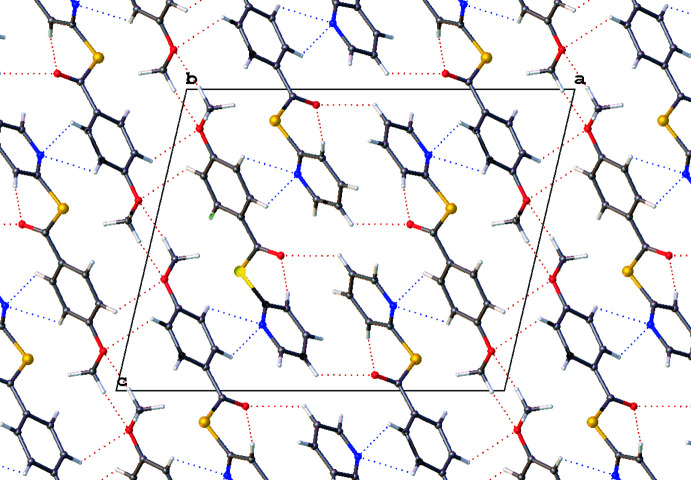
Hydrogen-bonding networks represented by dotted lines shown in a excerpt of the packing structure of **3** viewed normal to the *b* axis. Generated using *OLEX2*.

Compound **3** presents a multitude of non-classical hydrogen-bonding inter­actions, of the C—H⋯O_carbon­yl_ and the C—H⋯N_pyrid­yl_ type (Table 4 ▸, Fig. 8 ▸). The carbonyl O9 is linked by a bifurcated inter­action to C3-H and C5-H [*D*⋯*A* = 3.2566 (15) and 3.4270 (16) Å, respectively]. There is another bifurcated hydrogen-bond inter­action between the pyridine N1 and C11 and C12 [*D*⋯*A* = 3.3535 (17) and 3.4182 (16) Å, respectively], linking the mol­ecules head to tail. The meth­oxy groups form C17-H⋯O16 inter­actions [*D*⋯*A* = 3.4475 (17) Å], comprising a supra­molecular synthon linking two mol­ecules together. The meth­oxy oxygen O16 is further linked by a phenyl C14-H⋯O16 inter­action [*D*⋯*A* = 3.3340 (15) Å].

π–π stacking is evident in both **1** and **2**. Weak dimeric offset π–π stacking is observed in **1** with columns of anti-parallel non-inter­acting mol­ecules when viewed normal to (001) (Fig. 6 ▸). The closest centroid–centroid distance in **1** (C10–C15 to C10^i^–C15^i^ and N1–C6 to N1^i^–C6^i^ [symmetry transformation: (i) *x*, *y*, −1 + *z*; *x*, *y*, 1 + *z*] is 3.850 (3) Å with a slippages of 1.823 and 1.856 Å, respectively, and angles between planes of 0.0 (2)°. In **2**, π-stacking occurs only through phenyl ring pairs with the closest centroid–centroid distance being 3.8783 (11) Å, a slippage of 1.575 Å, and an angle between planes of 0.03 (9)°, as seen normal to the (011) plane. In **3** there is no relevant π–π stacking, with the closest centroid–centroid distance being 4.0847 (7) Å, with a slippage of 2.042 Å and an angle between the planes of 5.14 (6)°.

## 4. Database survey

A search in the Cambridge Structural Database (CSD, Version 5.43, update November 2022; Groom *et al.*, 2016 ▸) shows that no pyridine-substituted benzo­thio­ester structures are in the database. The unsubstituted *S*-phenyl benzo­thio­ate (CEFMOR; Belay *et al.*, 2012 ▸) is similar structurally to **1** with only slight ring-twisting differences. However, the packing is quite different with only weak dimeric offset π–π stacking present in **1**, with columns of anti-parallel non-inter­acting mol­ecules when viewed normal to (001). The distinct C—H⋯N inter­actions seen particularly in **3** do not exist in the phenyl homologue.

Several other phenyl benzo­thiol­ates, however, are in the database, including, (−)-*S*-phenyl 2-benzoyl­benzo­thio­ate (HOBREV; Takahashi *et al.*, 1998*a* ▸), (±)-*S*-phenyl 2-(*p*-tolyl­carbon­yl)benzo­thio­ate (HOBRUL; Takahashi *et al.*, 1998*a* ▸), (±)-*S*-phenyl 2-(*p*-chloro­phenyl­carbon­yl)benzo­thio­ate (HOBSAS; Takahashi *et al.*, 1998*a* ▸), *S*-phenyl-*p*-cyano­thio­benzoate (MEBDED; Ivanova *et al.*, 2006 ▸), *S*,*S*-diphenyl 2-bromo­benzene-1,3-bis­(carbo­thio­ate) (MOFQUV; Kathe­wad *et al.*, 2014 ▸), *S*-phenyl *o*-chloro­thio­benzoate (PEDHOV; Jovanovski *et al.*, 1993 ▸) and *S*-phenyl *o*-bromo­thio­benzoate (PEDHUB; Jovanovski *et al.*, 1993 ▸), *S*-phenyl 4-methyl-2-benzoyl­benzo­thio­ate (PUGXEU; Takahashi *et al.*, 1998*b* ▸; PUGXEU01; Takahashi *et al.*, 1998*a* ▸), *S* ^1^,*S* ^4^-diphenyl 2,5-bis­(di­phenyl­amino)­benzene-1,4-dicarbo­thio­ate (XETHAI; Shimizu *et al.*, 2016 ▸) and *S*-phenyl 4-meth­oxy­benzene­carbo­thio­ate (YAWYEC; El-Azab *et al.*, 2012 ▸; YAWYEC01; El-Azab & Abdel-Aziz, 2012 ▸).

## 5. Synthesis and crystallization

Compounds **1**, **2**, and **3** were synthesized following the reported procedure (Rao *et al.*, 2000 ▸). Briefly, the respective acyl chloride (1 eq., *ca* 0.2 *M*) in a solution of CH_2_Cl_2_ was added dropwise over 0.5 h to a stirring solution of 2-mercapto­pyridine (1 eq., *ca* 0.2 *M*) in CH_2_Cl_2_. The solution was left to stir for a further 2 h at room temperature. Throughout the addition processes, minor exotherms were noted, particularly for **1**. The solution was diluted with the same volume again of CH_2_Cl_2_, and the solution was washed with NaOH (2 *M*), water, brine, and the organic layer then dried (MgSO_4_). Excess solvent was removed under reduced pressure and the title compounds were purified in the following ways: for **1**, crystals were generated *via* hot recrystallization from ethyl acetate, and for **2** and **3**, crystals were generated *via* precipitation from diethyl ether and hexa­nes. Compound **1** was yielded in 69%, with yields for **2** and **3** comparable to those previously reported (Rao *et al.*, 2000 ▸).

^1^H NMR spectroscopic data matched previously reported synthesized compounds **2** and **3**. Whilst the synthesis of compound **1** has been reported previously, no characterization data has been reported for it (Perrin *et al.*, 2011 ▸). Below we present analytical data for **1**, and within the supporting information we have attached the appropriate spectra, Figs. S1–S3. We also present there the NMR spectra for **2** and **3**, to exhibit the electronic differences between the three compounds studied herein (Fig. S4).

Analytical data for **1**:

^1^H NMR (298 K, 400 MHz, CDCl_3_) δ = 8.66–8.68 (*m*, 1H), 8.31 (*d*, *J* = 8.9 Hz, 2H), 8.14 (*d*, *J* = 8.9 Hz, 2H), 7.77–7.81 (*m*, 1H), 7.68–7.70 (*m*, 1H), 7.33–7.37 (*m*, 1H); ^13^C{^1^H} NMR (298 K, 101 MHz, CDCl_3_): δ = 188.3, 150.9, 150.3, 141.3, 137.7, 130.9, 128.7, 124.3, 124.2 ppm; *R*_F_ = 0.58 (silica, EtOAc:C_6_H_14_ 1:1, UV); m.p. = 427–429 K. Multiple attempts have been made to obtain a mol­ecular ion peak *via* ESI–MS and all have been unsuccessful.

## 6. Refinement

Crystal data, data collection and structure refinement details are summarized in Table 5 ▸. Hydrogen atoms were positioned geometrically and refined isotropically using a riding model with C—H = 0.93–0.98 Å and *U*_iso_(H) = 1.2–1.5*U*_eq_(C).

## Supplementary Material

Crystal structure: contains datablock(s) 1, 2, 3, global. DOI: 10.1107/S2056989023001056/yz2028sup1.cif

Structure factors: contains datablock(s) 1. DOI: 10.1107/S2056989023001056/yz20281sup2.hkl

Structure factors: contains datablock(s) 2. DOI: 10.1107/S2056989023001056/yz20282sup3.hkl

Structure factors: contains datablock(s) 3. DOI: 10.1107/S2056989023001056/yz20283sup4.hkl

Supporting information file. DOI: 10.1107/S2056989023001056/yz20281sup5.cml

Supporting information file. DOI: 10.1107/S2056989023001056/yz20282sup6.cml

Supporting information file. DOI: 10.1107/S2056989023001056/yz20283sup7.cml

1H, 13C, and 1H-13C-HSQC NMR spectra of compound 1, along with overlayed aromatic regions of the 1H NMR spectra of compounds 1, 2, and 3. DOI: 10.1107/S2056989023001056/yz2028sup8.pdf

CCDC references: 2239845, 2239844, 2239843

Additional supporting information:  crystallographic information; 3D view; checkCIF report

## Figures and Tables

**Table 1:** Comparison of structural parameters (°)

	Plane|plane	Torsion angle C8—S1—C6—N1	Phenyl plane|plane C8—O9—S1—C6
**1**	56.97 (14)	128.6 (3)	6.00 (14)
**2**	57.51 (6)	120.11 (14)	5.08 (6)
**3**	65.94 (4)	75.84 (10)	10.28 (4)
CEFMOR	51.12 (1)	122.79 (1)	10.88 (2)

In CEFMOR, the torsion angle is defined by C1—S1—C8—C13.

**Table 2:** Hydrogen-bond geometry (Å, °) for **1** [Chem scheme1]

*D*—H⋯*A*	*D*—H	H⋯*A*	*D*⋯*A*	*D*—H⋯*A*
C3—H3⋯O18^i^	0.95	2.68	3.396 (5)	133
C4—H4⋯O9^ii^	0.95	2.46	3.283 (4)	145
C5—H5⋯O9^iii^	0.95	2.56	3.371 (5)	143
C12—H12⋯N1^iv^	0.95	2.49	3.315 (5)	145
C14—H14⋯O17^v^	0.95	2.77	3.359 (4)	121
C15—H15⋯O17^v^	0.95	2.66	3.312 (5)	127

Symmetry codes: (i) 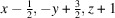 ; (ii) 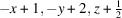 ; (iii) 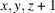 ; (iv) 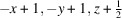 ; (v) 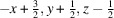 .

**Table 3:** Hydrogen-bond geometry (Å, °) for **2** [Chem scheme1]

*D*—H⋯*A*	*D*—H	H⋯*A*	*D*⋯*A*	*D*—H⋯*A*
C2—H2⋯N1^i^	0.95	2.70	3.355 (2)	126
C3—H3⋯O9^ii^	0.95	2.67	3.278 (2)	122
C4—H4⋯O9^ii^	0.95	2.75	3.316 (2)	119
C16—H16*B*⋯O9^iii^	0.98	2.64	3.460 (2)	142

Symmetry codes: (i) 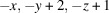 ; (ii) 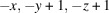 ; (iii) 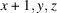 .

**Table 4:** Hydrogen-bond geometry (Å, °) for **3** [Chem scheme1]

*D*—H⋯*A*	*D*—H	H⋯*A*	*D*⋯*A*	*D*—H⋯*A*
C3—H3⋯O9^i^	0.95	2.65	3.2566 (15)	122
C5—H5⋯O9^ii^	0.95	2.49	3.4270 (16)	170
C11—H11⋯N1^iii^	0.95	2.69	3.3535 (17)	128
C12—H12⋯N1^iii^	0.95	2.84	3.4182 (16)	120
C14—H14⋯O16^iv^	0.95	2.67	3.3340 (15)	127
C17—H17*A*⋯O16^v^	0.98	2.63	3.4475 (17)	141

Symmetry codes: (i) 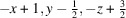 ; (ii) 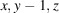 ; (iii) 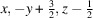 ; (iv) 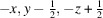 ; (v) 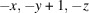 .

**Table 5:** Experimental details

	**1**	**2**	**3**
Crystal data			
Chemical formula	C_12_H_8_N_2_O_3_S	C_13_H_11_NOS	C_13_H_11_NO_2_S
*M*_r_	260.26	229.29	245.29
Crystal system, space group	Orthorhombic, *P* *n* *a*2_1_	Triclinic, *P* 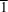	Monoclinic, *P*2_1_/*c*
Temperature (K)	100	100	100
*a*, *b*, *c* (Å)	23.0774 (11), 12.5622 (5), 3.8498 (2)	7.1775 (2), 9.1492 (3), 9.2832 (3)	16.4043 (6), 5.4939 (2), 13.0741 (4)
α, β, γ (°)	90, 90, 90	101.2966 (14), 108.4632 (13), 92.5673 (14)	90, 103.1748 (14), 90
*V* (Å^3^)	1116.07 (9)	563.28 (3)	1147.27 (7)
*Z*	4	2	4
Radiation type	Cu *K*α	Cu *K*α	Mo *K*α
μ (mm^−1^)	2.62	2.35	0.27
Crystal size (mm)	0.37 × 0.05 × 0.04	0.39 × 0.22 × 0.09	0.34 × 0.19 × 0.06

Data collection			
Diffractometer	Bruker APEXII Kappa Duo	Bruker APEXII Kappa Duo	Bruker D8 Quest ECO
Absorption correction	Multi-scan (*SADABS*; Krause *et al.*, 2015 ▸)	Multi-scan (*SADABS*; Krause *et al.*, 2015 ▸)	Multi-scan (*SADABS*; Krause *et al.*, 2015 ▸)
*T*_min_, *T*_max_	0.596, 0.753	0.641, 0.753	0.693, 0.746
No. of measured, independent and observed [*I* > 2σ(*I*)] reflections	8764, 1870, 1759	7937, 2106, 1958	19745, 3528, 2894
*R*_int_	0.062	0.036	0.035
(sin θ/λ)_max_ (Å^−1^)	0.609	0.610	0.716

Refinement			
*R*[*F* ^2^ > 2σ(*F* ^2^)], *wR*(*F* ^2^), *S*	0.043, 0.122, 1.04	0.042, 0.126, 1.12	0.036, 0.089, 1.03
No. of reflections	1870	2106	3528
No. of parameters	163	146	156
No. of restraints	1	0	0
H-atom treatment	H-atom parameters constrained	H-atom parameters constrained	H-atom parameters constrained
Δρ_max_, Δρ_min_ (e Å^−3^)	0.45, −0.26	0.33, −0.27	0.44, −0.34
Absolute structure	Flack *x* determined using 584 quotients [(*I* ^+^)−(*I* ^−^)]/[(*I* ^+^)+(*I* ^−^)] (Parsons *et al.*, 2013 ▸)	–	–
Absolute structure parameter	0.02 (3)	–	–

Computer programs: *APEX3* (Bruker, 2017 ▸), *APEX4* (Bruker, 2021 ▸), *SAINT* (Bruker, 2016 ▸), *SHELXT* (Sheldrick, 2015*a* ▸), *SHELXL* (Sheldrick, 2015*b* ▸) and *OLEX2* (Dolomanov *et al.*, 2009 ▸).

## supplementary crystallographic information

### S-(Pyridin-2-yl) 4-nitrobenzothioate (1) Crystal data

**Table:**

C_12_H_8_N_2_O_3_S	*D*_x_ = 1.549 Mg m^−^^3^
*M**_r_* = 260.26	Cu *K*α radiation, λ = 1.54178 Å
Orthorhombic, *P**n**a*2_1_	Cell parameters from 5322 reflections
*a* = 23.0774 (11) Å	θ = 3.8–69.3°
*b* = 12.5622 (5) Å	µ = 2.62 mm^−^^1^
*c* = 3.8498 (2) Å	*T* = 100 K
*V* = 1116.07 (9) Å^3^	Needle, clear colourless
*Z* = 4	0.37 × 0.05 × 0.04 mm
*F*(000) = 536	

### S-(Pyridin-2-yl) 4-nitrobenzothioate (1) Data collection

**Table:**

Bruker APEXII Kappa Duo diffractometer	1870 independent reflections
Radiation source: microfocus sealed X-ray tube, Incoatec Iµs	1759 reflections with *I* > 2σ(*I*)
Mirror optics monochromator	*R*_int_ = 0.062
Detector resolution: 8.33 pixels mm^-1^	θ_max_ = 69.8°, θ_min_ = 3.8°
ω and φ scans	*h* = −27→28
Absorption correction: multi-scan (SADABS; Krause *et al.*, 2015)	*k* = −15→15
*T*_min_ = 0.596, *T*_max_ = 0.753	*l* = −4→4
8764 measured reflections	

### S-(Pyridin-2-yl) 4-nitrobenzothioate (1) Refinement

**Table:**

Refinement on *F*^2^	Hydrogen site location: inferred from neighbouring sites
Least-squares matrix: full	H-atom parameters constrained
*R*[*F*^2^ > 2σ(*F*^2^)] = 0.043	*w* = 1/[σ^2^(*F*_o_^2^) + (0.0767*P*)^2^ + 0.6178*P*] where *P* = (*F*_o_^2^ + 2*F*_c_^2^)/3
*wR*(*F*^2^) = 0.122	(Δ/σ)_max_ < 0.001
*S* = 1.03	Δρ_max_ = 0.45 e Å^−^^3^
1870 reflections	Δρ_min_ = −0.26 e Å^−^^3^
163 parameters	Absolute structure: Flack *x* determined using 584 quotients [(*I*^+^)-(*I*^-^)]/[(*I*^+^)+(*I*^-^)] (Parsons *et al.*, 2013)
1 restraint	Absolute structure parameter: 0.02 (3)
Primary atom site location: dual	

### S-(Pyridin-2-yl) 4-nitrobenzothioate (1) Special details

**Table:**

Geometry. All esds (except the esd in the dihedral angle between two l.s. planes) are estimated using the full covariance matrix. The cell esds are taken into account individually in the estimation of esds in distances, angles and torsion angles; correlations between esds in cell parameters are only used when they are defined by crystal symmetry. An approximate (isotropic) treatment of cell esds is used for estimating esds involving l.s. planes.

### S-(Pyridin-2-yl) 4-nitrobenzothioate (1) Fractional atomic coordinates and isotropic or equivalent isotropic displacement parameters (Å^2^)

**Table:**

	*x*	*y*	*z*	*U*_iso_*/*U*_eq_	
C2	0.36818 (18)	0.8352 (3)	0.5521 (14)	0.0304 (9)	
H2	0.330987	0.825272	0.451494	0.037*	
C3	0.38137 (18)	0.9340 (3)	0.6888 (13)	0.0306 (9)	
H3	0.354398	0.990981	0.674849	0.037*	
C4	0.43473 (19)	0.9481 (3)	0.8464 (12)	0.0282 (9)	
H4	0.444798	1.014804	0.945957	0.034*	
C5	0.47344 (17)	0.8632 (3)	0.8569 (12)	0.0259 (8)	
H5	0.510275	0.869893	0.964831	0.031*	
C6	0.45619 (15)	0.7686 (3)	0.7039 (12)	0.0240 (8)	
C8	0.56755 (16)	0.7014 (3)	0.5347 (11)	0.0250 (9)	
C10	0.61366 (16)	0.6184 (3)	0.4955 (11)	0.0228 (8)	
C11	0.60607 (17)	0.5140 (3)	0.6121 (11)	0.0238 (8)	
H11	0.571383	0.494357	0.728517	0.029*	
C12	0.64936 (16)	0.4390 (3)	0.5575 (12)	0.0250 (8)	
H12	0.644736	0.367567	0.633914	0.030*	
C13	0.69883 (17)	0.4706 (3)	0.3907 (12)	0.0249 (8)	
C14	0.70817 (17)	0.5742 (3)	0.2745 (11)	0.0251 (9)	
H14	0.743200	0.593441	0.161129	0.030*	
C15	0.66456 (17)	0.6483 (3)	0.3299 (11)	0.0244 (9)	
H15	0.669537	0.719721	0.254326	0.029*	
N1	0.40512 (14)	0.7521 (2)	0.5536 (10)	0.0265 (7)	
N16	0.74430 (14)	0.3901 (2)	0.3212 (10)	0.0278 (8)	
O9	0.57457 (12)	0.79289 (18)	0.4455 (9)	0.0293 (7)	
O17	0.73740 (13)	0.3009 (2)	0.4467 (10)	0.0379 (8)	
O18	0.78579 (13)	0.4153 (2)	0.1448 (12)	0.0449 (10)	
S1	0.50105 (4)	0.65266 (6)	0.7155 (4)	0.0241 (3)	

### S-(Pyridin-2-yl) 4-nitrobenzothioate (1) Atomic displacement parameters (Å^2^)

**Table:**

	*U*^11^	*U*^22^	*U*^33^	*U*^12^	*U*^13^	*U*^23^
C2	0.0261 (19)	0.0317 (19)	0.033 (2)	0.0016 (15)	0.0009 (19)	0.0019 (19)
C3	0.036 (2)	0.0242 (17)	0.031 (2)	0.0077 (14)	0.0060 (19)	0.003 (2)
C4	0.038 (2)	0.0191 (16)	0.028 (2)	0.0014 (15)	0.0044 (17)	−0.0004 (16)
C5	0.030 (2)	0.0210 (16)	0.027 (2)	−0.0019 (14)	0.0007 (17)	0.0000 (16)
C6	0.0271 (17)	0.0168 (15)	0.028 (2)	−0.0001 (13)	0.0030 (17)	0.0025 (17)
C8	0.0270 (18)	0.0194 (17)	0.029 (2)	−0.0038 (13)	−0.0023 (17)	0.0008 (16)
C10	0.0294 (19)	0.0161 (15)	0.023 (2)	−0.0021 (13)	−0.0042 (15)	0.0006 (15)
C11	0.0268 (18)	0.0187 (15)	0.026 (2)	−0.0035 (13)	−0.0012 (15)	0.0011 (15)
C12	0.0303 (19)	0.0154 (15)	0.029 (2)	−0.0033 (13)	−0.0041 (18)	0.0031 (15)
C13	0.0268 (19)	0.0189 (16)	0.029 (2)	0.0020 (13)	−0.0041 (16)	0.0007 (16)
C14	0.0259 (18)	0.0209 (16)	0.029 (2)	−0.0060 (13)	−0.0003 (16)	−0.0007 (16)
C15	0.0285 (19)	0.0171 (16)	0.028 (2)	−0.0024 (13)	−0.0045 (16)	0.0015 (15)
N1	0.0308 (16)	0.0216 (14)	0.0270 (19)	−0.0026 (12)	0.0015 (15)	0.0007 (15)
N16	0.0279 (17)	0.0229 (15)	0.033 (2)	0.0003 (13)	−0.0030 (14)	−0.0024 (15)
O9	0.0343 (15)	0.0164 (12)	0.0371 (18)	−0.0024 (10)	0.0016 (12)	0.0055 (13)
O17	0.0376 (16)	0.0192 (13)	0.057 (2)	0.0038 (10)	−0.0016 (15)	0.0071 (14)
O18	0.0337 (16)	0.0310 (14)	0.070 (3)	0.0042 (12)	0.0145 (17)	0.0034 (17)
S1	0.0266 (5)	0.0147 (4)	0.0310 (5)	−0.0017 (3)	0.0025 (3)	0.0006 (4)

### S-(Pyridin-2-yl) 4-nitrobenzothioate (1) Geometric parameters (Å, º)

**Table:**

C2—H2	0.9500	C10—C11	1.397 (5)
C2—C3	1.382 (6)	C10—C15	1.388 (6)
C2—N1	1.348 (5)	C11—H11	0.9500
C3—H3	0.9500	C11—C12	1.389 (5)
C3—C4	1.384 (6)	C12—H12	0.9500
C4—H4	0.9500	C12—C13	1.369 (6)
C4—C5	1.392 (5)	C13—C14	1.393 (5)
C5—H5	0.9500	C13—N16	1.482 (5)
C5—C6	1.385 (5)	C14—H14	0.9500
C6—N1	1.329 (5)	C14—C15	1.388 (5)
C6—S1	1.787 (3)	C15—H15	0.9500
C8—C10	1.497 (5)	N16—O17	1.231 (4)
C8—O9	1.210 (4)	N16—O18	1.215 (5)
C8—S1	1.793 (4)		
			
C3—C2—H2	118.1	C10—C11—H11	120.1
N1—C2—H2	118.1	C12—C11—C10	119.9 (4)
N1—C2—C3	123.7 (4)	C12—C11—H11	120.1
C2—C3—H3	120.7	C11—C12—H12	120.9
C2—C3—C4	118.5 (3)	C13—C12—C11	118.3 (3)
C4—C3—H3	120.7	C13—C12—H12	120.9
C3—C4—H4	120.5	C12—C13—C14	123.4 (3)
C3—C4—C5	119.0 (4)	C12—C13—N16	118.5 (3)
C5—C4—H4	120.5	C14—C13—N16	118.0 (4)
C4—C5—H5	121.3	C13—C14—H14	121.1
C6—C5—C4	117.5 (4)	C15—C14—C13	117.7 (4)
C6—C5—H5	121.3	C15—C14—H14	121.1
C5—C6—S1	121.5 (3)	C10—C15—H15	119.9
N1—C6—C5	125.0 (3)	C14—C15—C10	120.2 (3)
N1—C6—S1	113.4 (3)	C14—C15—H15	119.9
C10—C8—S1	114.2 (3)	C6—N1—C2	116.2 (3)
O9—C8—C10	122.5 (4)	O17—N16—C13	117.3 (3)
O9—C8—S1	123.3 (3)	O18—N16—C13	118.8 (3)
C11—C10—C8	122.2 (3)	O18—N16—O17	123.9 (3)
C15—C10—C8	117.3 (3)	C6—S1—C8	102.01 (17)
C15—C10—C11	120.5 (3)		
			
C2—C3—C4—C5	1.0 (7)	C12—C13—N16—O17	−6.7 (6)
C3—C2—N1—C6	1.5 (7)	C12—C13—N16—O18	173.1 (4)
C3—C4—C5—C6	0.6 (7)	C13—C14—C15—C10	−0.1 (6)
C4—C5—C6—N1	−1.3 (7)	C14—C13—N16—O17	174.9 (4)
C4—C5—C6—S1	−177.6 (3)	C14—C13—N16—O18	−5.3 (6)
C5—C6—N1—C2	0.3 (7)	C15—C10—C11—C12	−1.0 (6)
C5—C6—S1—C8	−54.6 (4)	N1—C2—C3—C4	−2.1 (8)
C8—C10—C11—C12	177.5 (4)	N1—C6—S1—C8	128.6 (3)
C8—C10—C15—C14	−177.7 (4)	N16—C13—C14—C15	177.8 (4)
C10—C8—S1—C6	−177.2 (3)	O9—C8—C10—C11	177.3 (4)
C10—C11—C12—C13	0.4 (6)	O9—C8—C10—C15	−4.1 (6)
C11—C10—C15—C14	0.9 (6)	O9—C8—S1—C6	1.5 (4)
C11—C12—C13—C14	0.3 (6)	S1—C6—N1—C2	176.9 (3)
C11—C12—C13—N16	−178.0 (4)	S1—C8—C10—C11	−4.0 (5)
C12—C13—C14—C15	−0.5 (6)	S1—C8—C10—C15	174.6 (3)

### S-(Pyridin-2-yl) 4-nitrobenzothioate (1) Hydrogen-bond geometry (Å, º)

**Table:**

*D*—H···*A*	*D*—H	H···*A*	*D*···*A*	*D*—H···*A*
C3—H3···O18^i^	0.95	2.68	3.396 (5)	133
C4—H4···O9^ii^	0.95	2.46	3.283 (4)	145
C5—H5···O9^iii^	0.95	2.56	3.371 (5)	143
C12—H12···N1^iv^	0.95	2.49	3.315 (5)	145
C14—H14···O17^v^	0.95	2.77	3.359 (4)	121
C15—H15···O17^v^	0.95	2.66	3.312 (5)	127

Symmetry codes: (i) *x*−1/2, −*y*+3/2, *z*+1; (ii) −*x*+1, −*y*+2, *z*+1/2; (iii) *x*, *y*, *z*+1; (iv) −*x*+1, −*y*+1, *z*+1/2; (v) −*x*+3/2, *y*+1/2, *z*−1/2.

### S-(Pyridin-2-yl) 4-methylbenzothioate (2) Crystal data

**Table:**

C_13_H_11_NOS	*Z* = 2
*M**_r_* = 229.29	*F*(000) = 240
Triclinic, *P*1	*D*_x_ = 1.352 Mg m^−^^3^
*a* = 7.1775 (2) Å	Cu *K*α radiation, λ = 1.54178 Å
*b* = 9.1492 (3) Å	Cell parameters from 5814 reflections
*c* = 9.2832 (3) Å	θ = 5.0–70.1°
α = 101.2966 (14)°	µ = 2.35 mm^−^^1^
β = 108.4632 (13)°	*T* = 100 K
γ = 92.5673 (14)°	Plate, clear colourless
*V* = 563.28 (3) Å^3^	0.39 × 0.22 × 0.09 mm

### S-(Pyridin-2-yl) 4-methylbenzothioate (2) Data collection

**Table:**

Bruker APEXII Kappa Duo diffractometer	2106 independent reflections
Radiation source: microfocus sealed X-ray tube, Incoatec Iµs	1958 reflections with *I* > 2σ(*I*)
Mirror optics monochromator	*R*_int_ = 0.036
Detector resolution: 8.33 pixels mm^-1^	θ_max_ = 70.0°, θ_min_ = 5.0°
ω and φ scans	*h* = −8→8
Absorption correction: multi-scan (SADABS; Krause *et al.*, 2015)	*k* = −10→10
*T*_min_ = 0.641, *T*_max_ = 0.753	*l* = −11→11
7937 measured reflections	

### S-(Pyridin-2-yl) 4-methylbenzothioate (2) Refinement

**Table:**

Refinement on *F*^2^	Primary atom site location: dual
Least-squares matrix: full	Hydrogen site location: inferred from neighbouring sites
*R*[*F*^2^ > 2σ(*F*^2^)] = 0.042	H-atom parameters constrained
*wR*(*F*^2^) = 0.126	*w* = 1/[σ^2^(*F*_o_^2^) + (0.0776*P*)^2^ + 0.2152*P*] where *P* = (*F*_o_^2^ + 2*F*_c_^2^)/3
*S* = 1.12	(Δ/σ)_max_ = 0.001
2106 reflections	Δρ_max_ = 0.33 e Å^−^^3^
146 parameters	Δρ_min_ = −0.27 e Å^−^^3^
0 restraints	

### S-(Pyridin-2-yl) 4-methylbenzothioate (2) Special details

**Table:**

Geometry. All esds (except the esd in the dihedral angle between two l.s. planes) are estimated using the full covariance matrix. The cell esds are taken into account individually in the estimation of esds in distances, angles and torsion angles; correlations between esds in cell parameters are only used when they are defined by crystal symmetry. An approximate (isotropic) treatment of cell esds is used for estimating esds involving l.s. planes.

### S-(Pyridin-2-yl) 4-methylbenzothioate (2) Fractional atomic coordinates and isotropic or equivalent isotropic displacement parameters (Å^2^)

**Table:**

	*x*	*y*	*z*	*U*_iso_*/*U*_eq_	
S1	0.49730 (6)	0.81629 (5)	0.54572 (5)	0.02575 (19)	
N1	0.1173 (2)	0.85006 (17)	0.44707 (17)	0.0239 (3)	
C2	−0.0761 (3)	0.8008 (2)	0.3793 (2)	0.0250 (4)	
H2	−0.167830	0.872979	0.368508	0.030*	
C3	−0.1488 (3)	0.6502 (2)	0.3242 (2)	0.0245 (4)	
H3	−0.286999	0.620400	0.278163	0.029*	
C4	−0.0162 (3)	0.5439 (2)	0.3376 (2)	0.0241 (4)	
H4	−0.061528	0.439922	0.300499	0.029*	
C5	0.1838 (3)	0.5928 (2)	0.4062 (2)	0.0237 (4)	
H5	0.279047	0.523231	0.416127	0.028*	
C6	0.2416 (3)	0.7455 (2)	0.4599 (2)	0.0219 (4)	
C8	0.5659 (3)	0.73280 (19)	0.7124 (2)	0.0216 (4)	
O9	0.44856 (19)	0.65460 (16)	0.74143 (15)	0.0294 (3)	
C10	0.7791 (3)	0.76691 (19)	0.8087 (2)	0.0218 (4)	
C11	0.8476 (3)	0.6978 (2)	0.9337 (2)	0.0241 (4)	
H11	0.757864	0.634654	0.957347	0.029*	
C12	1.0455 (3)	0.7206 (2)	1.0234 (2)	0.0250 (4)	
H12	1.090277	0.672879	1.108358	0.030*	
C13	1.1805 (3)	0.8126 (2)	0.9914 (2)	0.0247 (4)	
C14	1.1111 (3)	0.8823 (2)	0.8667 (2)	0.0255 (4)	
H14	1.201135	0.945560	0.843497	0.031*	
C15	0.9125 (3)	0.8605 (2)	0.7760 (2)	0.0229 (4)	
H15	0.867412	0.909236	0.691895	0.028*	
C16	1.3959 (3)	0.8344 (2)	1.0883 (2)	0.0313 (5)	
H16A	1.433901	0.939493	1.144431	0.047*	
H16B	1.475338	0.808356	1.020416	0.047*	
H16C	1.418740	0.769623	1.163247	0.047*	

### S-(Pyridin-2-yl) 4-methylbenzothioate (2) Atomic displacement parameters (Å^2^)

**Table:**

	*U*^11^	*U*^22^	*U*^33^	*U*^12^	*U*^13^	*U*^23^
S1	0.0200 (3)	0.0285 (3)	0.0265 (3)	−0.00191 (19)	0.00180 (19)	0.0118 (2)
N1	0.0244 (8)	0.0223 (8)	0.0238 (7)	0.0017 (6)	0.0060 (6)	0.0056 (6)
C2	0.0217 (9)	0.0264 (10)	0.0276 (9)	0.0063 (7)	0.0068 (7)	0.0090 (7)
C3	0.0213 (9)	0.0292 (10)	0.0229 (9)	0.0014 (7)	0.0065 (7)	0.0074 (7)
C4	0.0274 (9)	0.0224 (9)	0.0199 (8)	−0.0008 (7)	0.0051 (7)	0.0042 (7)
C5	0.0245 (9)	0.0239 (9)	0.0217 (8)	0.0056 (7)	0.0058 (7)	0.0053 (7)
C6	0.0212 (8)	0.0241 (9)	0.0197 (8)	0.0018 (7)	0.0051 (7)	0.0063 (7)
C8	0.0221 (9)	0.0200 (9)	0.0212 (8)	0.0014 (7)	0.0062 (7)	0.0027 (7)
O9	0.0229 (7)	0.0362 (8)	0.0285 (7)	−0.0035 (6)	0.0055 (5)	0.0122 (6)
C10	0.0217 (9)	0.0201 (9)	0.0216 (8)	0.0020 (7)	0.0061 (7)	0.0019 (7)
C11	0.0252 (9)	0.0238 (9)	0.0237 (9)	0.0019 (7)	0.0088 (7)	0.0056 (7)
C12	0.0259 (9)	0.0269 (10)	0.0217 (9)	0.0051 (7)	0.0060 (7)	0.0070 (7)
C13	0.0228 (9)	0.0259 (9)	0.0219 (8)	0.0021 (7)	0.0052 (7)	0.0010 (7)
C14	0.0233 (9)	0.0248 (9)	0.0265 (9)	−0.0021 (7)	0.0067 (7)	0.0045 (7)
C15	0.0226 (9)	0.0216 (9)	0.0224 (9)	0.0011 (7)	0.0043 (7)	0.0051 (7)
C16	0.0231 (10)	0.0379 (11)	0.0284 (10)	0.0018 (8)	0.0029 (8)	0.0068 (8)

### S-(Pyridin-2-yl) 4-methylbenzothioate (2) Geometric parameters (Å, º)

**Table:**

S1—C6	1.7853 (18)	C10—C11	1.394 (3)
S1—C8	1.7981 (18)	C10—C15	1.398 (3)
N1—C2	1.344 (2)	C11—H11	0.9500
N1—C6	1.333 (2)	C11—C12	1.383 (3)
C2—H2	0.9500	C12—H12	0.9500
C2—C3	1.387 (3)	C12—C13	1.394 (3)
C3—H3	0.9500	C13—C14	1.395 (3)
C3—C4	1.386 (3)	C13—C16	1.503 (2)
C4—H4	0.9500	C14—H14	0.9500
C4—C5	1.385 (3)	C14—C15	1.389 (2)
C5—H5	0.9500	C15—H15	0.9500
C5—C6	1.385 (3)	C16—H16A	0.9800
C8—O9	1.208 (2)	C16—H16B	0.9800
C8—C10	1.490 (2)	C16—H16C	0.9800
			
C6—S1—C8	100.57 (8)	C15—C10—C8	122.97 (16)
C6—N1—C2	116.56 (16)	C10—C11—H11	119.8
N1—C2—H2	118.3	C12—C11—C10	120.32 (17)
N1—C2—C3	123.44 (16)	C12—C11—H11	119.8
C3—C2—H2	118.3	C11—C12—H12	119.5
C2—C3—H3	120.6	C11—C12—C13	121.07 (17)
C4—C3—C2	118.82 (16)	C13—C12—H12	119.5
C4—C3—H3	120.6	C12—C13—C14	118.44 (17)
C3—C4—H4	120.8	C12—C13—C16	120.50 (17)
C5—C4—C3	118.49 (17)	C14—C13—C16	121.06 (17)
C5—C4—H4	120.8	C13—C14—H14	119.5
C4—C5—H5	120.8	C15—C14—C13	121.01 (17)
C6—C5—C4	118.36 (16)	C15—C14—H14	119.5
C6—C5—H5	120.8	C10—C15—H15	120.0
N1—C6—S1	114.99 (14)	C14—C15—C10	119.94 (16)
N1—C6—C5	124.31 (16)	C14—C15—H15	120.0
C5—C6—S1	120.64 (14)	C13—C16—H16A	109.5
O9—C8—S1	122.42 (14)	C13—C16—H16B	109.5
O9—C8—C10	123.49 (16)	C13—C16—H16C	109.5
C10—C8—S1	114.09 (12)	H16A—C16—H16B	109.5
C11—C10—C8	117.77 (16)	H16A—C16—H16C	109.5
C11—C10—C15	119.22 (16)	H16B—C16—H16C	109.5
			
S1—C8—C10—C11	−175.48 (13)	C8—S1—C6—C5	−62.55 (16)
S1—C8—C10—C15	2.5 (2)	C8—C10—C11—C12	177.48 (16)
N1—C2—C3—C4	−0.8 (3)	C8—C10—C15—C14	−177.17 (16)
C2—N1—C6—S1	178.32 (12)	O9—C8—C10—C11	3.7 (3)
C2—N1—C6—C5	1.1 (3)	O9—C8—C10—C15	−178.40 (17)
C2—C3—C4—C5	0.3 (3)	C10—C11—C12—C13	0.0 (3)
C3—C4—C5—C6	0.8 (3)	C11—C10—C15—C14	0.7 (3)
C4—C5—C6—S1	−178.64 (13)	C11—C12—C13—C14	0.4 (3)
C4—C5—C6—N1	−1.6 (3)	C11—C12—C13—C16	−178.99 (17)
C6—S1—C8—O9	−1.13 (17)	C12—C13—C14—C15	−0.2 (3)
C6—S1—C8—C10	178.01 (12)	C13—C14—C15—C10	−0.4 (3)
C6—N1—C2—C3	0.1 (3)	C15—C10—C11—C12	−0.5 (3)
C8—S1—C6—N1	120.11 (14)	C16—C13—C14—C15	179.19 (16)

### S-(Pyridin-2-yl) 4-methylbenzothioate (2) Hydrogen-bond geometry (Å, º)

**Table:**

*D*—H···*A*	*D*—H	H···*A*	*D*···*A*	*D*—H···*A*
C2—H2···N1^i^	0.95	2.70	3.355 (2)	126
C3—H3···O9^ii^	0.95	2.67	3.278 (2)	122
C4—H4···O9^ii^	0.95	2.75	3.316 (2)	119
C16—H16*B*···O9^iii^	0.98	2.64	3.460 (2)	142

Symmetry codes: (i) −*x*, −*y*+2, −*z*+1; (ii) −*x*, −*y*+1, −*z*+1; (iii) *x*+1, *y*, *z*.

### S-(Pyridin-2-yl) 4-methoxybenzothioate (3) Crystal data

**Table:**

C_13_H_11_NO_2_S	*F*(000) = 512
*M**_r_* = 245.29	*D*_x_ = 1.420 Mg m^−^^3^
Monoclinic, *P*2_1_/*c*	Mo *K*α radiation, λ = 0.71073 Å
*a* = 16.4043 (6) Å	Cell parameters from 9968 reflections
*b* = 5.4939 (2) Å	θ = 2.6–30.6°
*c* = 13.0741 (4) Å	µ = 0.27 mm^−^^1^
β = 103.1748 (14)°	*T* = 100 K
*V* = 1147.27 (7) Å^3^	Plate, clear colourless
*Z* = 4	0.34 × 0.19 × 0.06 mm

### S-(Pyridin-2-yl) 4-methoxybenzothioate (3) Data collection

**Table:**

Bruker D8 Quest ECO diffractometer	3528 independent reflections
Radiation source: sealed X-ray tube, Siemens, KFF Mo 2K -90 C	2894 reflections with *I* > 2σ(*I*)
Graphite monochromator	*R*_int_ = 0.035
Detector resolution: 5.12 pixels mm^-1^	θ_max_ = 30.6°, θ_min_ = 2.6°
ω and φ scans	*h* = −23→23
Absorption correction: multi-scan (SADABS; Krause *et al.*, 2015)	*k* = −7→7
*T*_min_ = 0.693, *T*_max_ = 0.746	*l* = −18→18
19745 measured reflections	

### S-(Pyridin-2-yl) 4-methoxybenzothioate (3) Refinement

**Table:**

Refinement on *F*^2^	Hydrogen site location: inferred from neighbouring sites
Least-squares matrix: full	H-atom parameters constrained
*R*[*F*^2^ > 2σ(*F*^2^)] = 0.036	*w* = 1/[σ^2^(*F*_o_^2^) + (0.034*P*)^2^ + 0.7686*P*] where *P* = (*F*_o_^2^ + 2*F*_c_^2^)/3
*wR*(*F*^2^) = 0.089	(Δ/σ)_max_ = 0.001
*S* = 1.03	Δρ_max_ = 0.44 e Å^−^^3^
3528 reflections	Δρ_min_ = −0.34 e Å^−^^3^
156 parameters	Extinction correction: SHELXL (Sheldrick 2015b), Fc^*^=kFc[1+0.001xFc^2^λ^3^/sin(2θ)]^-1/4^
0 restraints	Extinction coefficient: 0.0056 (14)
Primary atom site location: dual	

### S-(Pyridin-2-yl) 4-methoxybenzothioate (3) Special details

**Table:**

Geometry. All esds (except the esd in the dihedral angle between two l.s. planes) are estimated using the full covariance matrix. The cell esds are taken into account individually in the estimation of esds in distances, angles and torsion angles; correlations between esds in cell parameters are only used when they are defined by crystal symmetry. An approximate (isotropic) treatment of cell esds is used for estimating esds involving l.s. planes.

### S-(Pyridin-2-yl) 4-methoxybenzothioate (3) Fractional atomic coordinates and isotropic or equivalent isotropic displacement parameters (Å^2^)

**Table:**

	*x*	*y*	*z*	*U*_iso_*/*U*_eq_	
N1	0.33724 (7)	0.3844 (2)	0.78131 (9)	0.0186 (2)	
C2	0.40114 (8)	0.3820 (2)	0.86600 (10)	0.0191 (2)	
H2	0.405275	0.511908	0.914884	0.023*	
C3	0.46148 (8)	0.2013 (2)	0.88640 (9)	0.0175 (2)	
H3	0.505834	0.208357	0.947409	0.021*	
C4	0.45592 (8)	0.0107 (2)	0.81631 (10)	0.0186 (2)	
H4	0.496434	−0.115916	0.828212	0.022*	
C5	0.38991 (8)	0.0072 (2)	0.72793 (10)	0.0177 (2)	
H5	0.383763	−0.122007	0.678476	0.021*	
C6	0.33342 (7)	0.1989 (2)	0.71454 (9)	0.0155 (2)	
S1	0.24650 (2)	0.19535 (6)	0.60431 (2)	0.02004 (9)	
C8	0.27774 (7)	0.4257 (2)	0.52399 (9)	0.0141 (2)	
O9	0.34259 (6)	0.53792 (18)	0.55168 (7)	0.0197 (2)	
C10	0.21680 (7)	0.4661 (2)	0.42271 (9)	0.0136 (2)	
C11	0.22903 (7)	0.6656 (2)	0.36245 (9)	0.0158 (2)	
H11	0.274363	0.773119	0.388522	0.019*	
C12	0.17643 (8)	0.7112 (2)	0.26502 (9)	0.0166 (2)	
H12	0.185178	0.849052	0.224987	0.020*	
C13	0.11069 (7)	0.5514 (2)	0.22707 (9)	0.0147 (2)	
C14	0.09620 (7)	0.3538 (2)	0.28750 (10)	0.0173 (2)	
H14	0.050131	0.248509	0.261957	0.021*	
C15	0.14888 (7)	0.3111 (2)	0.38464 (9)	0.0162 (2)	
H15	0.138956	0.176179	0.425619	0.019*	
O16	0.05718 (5)	0.57034 (18)	0.13098 (7)	0.01853 (19)	
C17	0.07223 (9)	0.7626 (3)	0.06401 (11)	0.0237 (3)	
H17A	0.031733	0.752011	−0.003703	0.036*	
H17B	0.129116	0.747907	0.052882	0.036*	
H17C	0.066163	0.919806	0.096929	0.036*	

### S-(Pyridin-2-yl) 4-methoxybenzothioate (3) Atomic displacement parameters (Å^2^)

**Table:**

	*U*^11^	*U*^22^	*U*^33^	*U*^12^	*U*^13^	*U*^23^
N1	0.0185 (5)	0.0166 (5)	0.0193 (5)	0.0021 (4)	0.0014 (4)	−0.0013 (4)
C2	0.0205 (6)	0.0179 (6)	0.0174 (5)	0.0009 (5)	0.0012 (4)	−0.0041 (5)
C3	0.0163 (5)	0.0195 (6)	0.0149 (5)	−0.0002 (5)	−0.0002 (4)	0.0020 (5)
C4	0.0174 (5)	0.0164 (6)	0.0214 (6)	0.0027 (4)	0.0030 (4)	0.0025 (5)
C5	0.0200 (6)	0.0157 (5)	0.0172 (5)	−0.0008 (5)	0.0038 (4)	−0.0015 (4)
C6	0.0151 (5)	0.0162 (5)	0.0140 (5)	−0.0023 (4)	0.0011 (4)	0.0020 (4)
S1	0.01791 (15)	0.02262 (17)	0.01665 (15)	−0.00707 (12)	−0.00218 (10)	0.00527 (12)
C8	0.0151 (5)	0.0139 (5)	0.0134 (5)	0.0000 (4)	0.0035 (4)	−0.0013 (4)
O9	0.0170 (4)	0.0229 (5)	0.0179 (4)	−0.0067 (4)	0.0011 (3)	−0.0003 (4)
C10	0.0133 (5)	0.0140 (5)	0.0134 (5)	−0.0004 (4)	0.0030 (4)	−0.0009 (4)
C11	0.0158 (5)	0.0156 (5)	0.0155 (5)	−0.0033 (4)	0.0024 (4)	−0.0016 (4)
C12	0.0192 (5)	0.0141 (5)	0.0162 (5)	−0.0011 (4)	0.0033 (4)	0.0015 (4)
C13	0.0126 (5)	0.0173 (6)	0.0139 (5)	0.0026 (4)	0.0023 (4)	−0.0010 (4)
C14	0.0136 (5)	0.0205 (6)	0.0168 (5)	−0.0040 (4)	0.0017 (4)	−0.0005 (4)
C15	0.0159 (5)	0.0172 (5)	0.0151 (5)	−0.0038 (4)	0.0027 (4)	0.0009 (4)
O16	0.0159 (4)	0.0233 (5)	0.0147 (4)	0.0006 (3)	−0.0002 (3)	0.0026 (3)
C17	0.0278 (7)	0.0222 (6)	0.0180 (6)	0.0029 (5)	−0.0013 (5)	0.0048 (5)

### S-(Pyridin-2-yl) 4-methoxybenzothioate (3) Geometric parameters (Å, º)

**Table:**

N1—C2	1.3401 (16)	C10—C15	1.4007 (16)
N1—C6	1.3342 (16)	C11—H11	0.9500
C2—H2	0.9500	C11—C12	1.3888 (16)
C2—C3	1.3841 (18)	C12—H12	0.9500
C3—H3	0.9500	C12—C13	1.3914 (17)
C3—C4	1.3810 (18)	C13—C14	1.3943 (17)
C4—H4	0.9500	C13—O16	1.3627 (14)
C4—C5	1.3920 (17)	C14—H14	0.9500
C5—H5	0.9500	C14—C15	1.3837 (16)
C5—C6	1.3873 (17)	C15—H15	0.9500
C6—S1	1.7815 (12)	O16—C17	1.4289 (16)
S1—C8	1.7924 (12)	C17—H17A	0.9800
C8—O9	1.2112 (14)	C17—H17B	0.9800
C8—C10	1.4828 (16)	C17—H17C	0.9800
C10—C11	1.3908 (17)		
			
C6—N1—C2	116.38 (11)	C10—C11—H11	119.3
N1—C2—H2	118.1	C12—C11—C10	121.46 (11)
N1—C2—C3	123.83 (12)	C12—C11—H11	119.3
C3—C2—H2	118.1	C11—C12—H12	120.6
C2—C3—H3	120.7	C11—C12—C13	118.84 (11)
C4—C3—C2	118.68 (11)	C13—C12—H12	120.6
C4—C3—H3	120.7	C12—C13—C14	120.49 (11)
C3—C4—H4	120.6	O16—C13—C12	124.37 (11)
C3—C4—C5	118.84 (12)	O16—C13—C14	115.14 (11)
C5—C4—H4	120.6	C13—C14—H14	120.0
C4—C5—H5	121.1	C15—C14—C13	120.05 (11)
C6—C5—C4	117.73 (12)	C15—C14—H14	120.0
C6—C5—H5	121.1	C10—C15—H15	119.9
N1—C6—C5	124.54 (11)	C14—C15—C10	120.18 (11)
N1—C6—S1	116.57 (9)	C14—C15—H15	119.9
C5—C6—S1	118.83 (9)	C13—O16—C17	117.16 (10)
C6—S1—C8	100.56 (6)	O16—C17—H17A	109.5
O9—C8—S1	122.22 (9)	O16—C17—H17B	109.5
O9—C8—C10	123.94 (11)	O16—C17—H17C	109.5
C10—C8—S1	113.84 (8)	H17A—C17—H17B	109.5
C11—C10—C8	117.96 (10)	H17A—C17—H17C	109.5
C11—C10—C15	118.93 (11)	H17B—C17—H17C	109.5
C15—C10—C8	123.10 (11)		
			
N1—C2—C3—C4	−0.4 (2)	C8—C10—C11—C12	177.74 (11)
N1—C6—S1—C8	75.84 (10)	C8—C10—C15—C14	−177.44 (12)
C2—N1—C6—C5	0.65 (19)	O9—C8—C10—C11	−9.07 (18)
C2—N1—C6—S1	177.79 (10)	O9—C8—C10—C15	169.83 (12)
C2—C3—C4—C5	−0.04 (19)	C10—C11—C12—C13	−0.62 (18)
C3—C4—C5—C6	0.72 (19)	C11—C10—C15—C14	1.45 (18)
C4—C5—C6—N1	−1.07 (19)	C11—C12—C13—C14	2.22 (18)
C4—C5—C6—S1	−178.15 (9)	C11—C12—C13—O16	−177.02 (11)
C5—C6—S1—C8	−106.85 (11)	C12—C13—C14—C15	−1.98 (19)
C6—N1—C2—C3	0.1 (2)	C12—C13—O16—C17	2.44 (17)
C6—S1—C8—O9	−1.13 (12)	C13—C14—C15—C10	0.12 (19)
C6—S1—C8—C10	179.52 (9)	C14—C13—O16—C17	−176.83 (11)
S1—C8—C10—C11	170.26 (9)	C15—C10—C11—C12	−1.20 (18)
S1—C8—C10—C15	−10.84 (15)	O16—C13—C14—C15	177.32 (11)

### S-(Pyridin-2-yl) 4-methoxybenzothioate (3) Hydrogen-bond geometry (Å, º)

**Table:**

*D*—H···*A*	*D*—H	H···*A*	*D*···*A*	*D*—H···*A*
C3—H3···O9^i^	0.95	2.65	3.2566 (15)	122
C5—H5···O9^ii^	0.95	2.49	3.4270 (16)	170
C11—H11···N1^iii^	0.95	2.69	3.3535 (17)	128
C12—H12···N1^iii^	0.95	2.84	3.4182 (16)	120
C14—H14···O16^iv^	0.95	2.67	3.3340 (15)	127
C17—H17*A*···O16^v^	0.98	2.63	3.4475 (17)	141

Symmetry codes: (i) −*x*+1, *y*−1/2, −*z*+3/2; (ii) *x*, *y*−1, *z*; (iii) *x*, −*y*+3/2, *z*−1/2; (iv) −*x*, *y*−1/2, −*z*+1/2; (v) −*x*, −*y*+1, −*z*.

## References

[bb1] Adler, D. A., Longo, F. R., Finarelli, J. D., Goldmacher, J., Assour, J. & Korsakoff, L. (1967). *J. Org. Chem.* **32**, 476–476.

[bb2] Albert, A. & Barlin, G. B. (1959). *J. Chem. Soc.* pp. 2384–2396.

[bb3] Araki, M., Sakata, S., Takei, H. & Mukaiyama, T. (1974). *Bull. Chem. Soc. Jpn*, **47**, 1777–1780.

[bb4] Belay, Y. H., Kinfe, H. H. & Muller, A. (2012). *Acta Cryst.* E**68**, o2825. 10.1107/S1600536812037142 PMC3470187 23125631

[bb5] Bruker (2016). *SAINT*. Bruker AXS Inc., Madison, Wisconsin, USA.

[bb6] Bruker (2017). *APEX3.* Bruker AXS Inc., Madison, Wisconsin, USA.

[bb7] Bruker (2021). *APEX4*. Bruker AXS Inc., Madison, Wisconsin, USA.

[bb8] Dolomanov, O. V., Bourhis, L. J., Gildea, R. J., Howard, J. A. K. & Puschmann, H. (2009). *J. Appl. Cryst.* **42**, 339–341.

[bb9] El-Azab, A. S. & Abdel-Aziz, A. A.-M. (2012). *Phosphorus Sulfur Silicon*, **187**, 1046–1055.

[bb10] El-Azab, A. S., Abdel-Aziz, A. A.-M., El-Subbagh, H. I., Chantrapromma, S. & Fun, H.-K. (2012). *Acta Cryst.* E**68**, o1074–o1075. 10.1107/S1600536812005454 PMC3344030 22589939

[bb11] Groom, C. R., Bruno, I. J., Lightfoot, M. P. & Ward, S. C. (2016). *Acta Cryst.* B**72**, 171–179. 10.1107/S2052520616003954 PMC4822653 27048719

[bb12] Hiroto, S., Miyake, Y. & Shinokubo, H. (2017). *Chem. Rev.* **117**, 2910–3043. 10.1021/acs.chemrev.6b00427 27709907

[bb13] Ivanova, B. B., Arnaudov, M. G., Sheldrick, W. S. & Mayer-Figge, H. (2006). *Acta Cryst.* E**62**, o3–o4.

[bb14] Jovanovski, G., Soptrajanov, B., Kaitner, B. & Prangova, L. (1993). *J. Crystallogr. Spectrosc. Res.* **23**, 49–53.

[bb15] Kathewad, N. V., Pal, S. & Khan, S. (2014). Private Communication (refcode MOFQUV). CCDC, Cambridge, England.

[bb16] Krause, L., Herbst-Irmer, R., Sheldrick, G. M. & Stalke, D. (2015). *J. Appl. Cryst.* **48**, 3–10. 10.1107/S1600576714022985 PMC4453166 26089746

[bb17] Laakso, J., Rosser, G. A., Szíjjártó, C., Beeby, A. & Borbas, K. E. (2012). *Inorg. Chem.* **51**, 10366–10374. 10.1021/ic3015354 22978627

[bb18] Lee, C.-H., Li, F., Iwamoto, K., Dadok, J., Bothner-By, A. A. & Lindsey, J. S. (1995). *Tetrahedron*, **51**, 11645–11672.

[bb19] Lee, J. (2020). *Bull. Korean Chem. Soc.* **41**, 735–747.

[bb20] Lindsey, J. S. (2010). *Acc. Chem. Res.* **43**, 300–311. 10.1021/ar900212t 19863076

[bb21] Lindsey, J. S., Hsu, H. C. & Schreiman, I. C. (1986). *Tetrahedron Lett.* **27**, 4969–4970.

[bb22] McDaniel, D. H. & Brown, H. C. (1958). *J. Org. Chem.* **23**, 420–427.

[bb23] Nicolaou, K. C., Claremon, D. A. & Papahatjis, D. P. (1981). *Tetrahedron Lett.* **22**, 4647–4650.

[bb24] Parsons, S., Flack, H. D. & Wagner, T. (2013). *Acta Cryst.* B**69**, 249–259. 10.1107/S2052519213010014 PMC3661305 23719469

[bb25] Perrin, M. L., Prins, F., Martin, C. A., Shaikh, A. J., Eelkema, R., van Esch, J. H., Briza, T., Kaplanek, R., Kral, V., van Ruitenbeek, J. M., van der Zant, H. S. J. & Dulić, D. (2011). *Angew. Chem. Int. Ed.* **50**, 11223–11226. 10.1002/anie.201104757 21957060

[bb26] Ra, D., Gauger, K. A., Muthukumaran, K., Balasubramanian, T., Chandrashaker, V., Taniguchi, M., Yu, Z., Talley, D. C., Ehudin, M. Ptaszek, M. & Lindsey J. S. (2015). *J. Porphyrins Phthalocyanines*, **19**, 547–572 10.1142/S1088424615500042 PMC4669101 26640361

[bb27] Rao, P. D., Littler, B. J., Geier, G. R. III & Lindsey, J. S. (2000). *J. Org. Chem.* **65**, 1084–1092. 10.1021/jo9915473 10814057

[bb28] Sample, H. C. & Senge, M. O. (2021). *Eur. J. Org. Chem.* **2021**, 7–42. 10.1002/ejoc.202001183 PMC7821298 33519299

[bb29] Senge, M. O. (2011). *Chem. Commun.* **47**, 1943–1960. 10.1039/c0cc03984e 21218237

[bb30] Senge, M. O., Sergeeva, N. N. & Hale, K. J. (2021). *Chem. Soc. Rev.* **50**, 4730–4789. 10.1039/c7cs00719a 33623938

[bb31] Sheldrick, G. M. (2015*a*). *Acta Cryst.* A**71**, 3–8.

[bb32] Sheldrick, G. M. (2015*b*). *Acta Cryst.* C**71**, 3–8.

[bb33] Shimizu, M., Fukui, H., Natakani, M. & Sakaguchi, H. (2016). *Eur. J. Org. Chem.* **2016**, 5950–5956.

[bb34] Takahashi, M., Fujita, T., Watanabe, S. & Sakamoto, M. (1998*b*). *J. Chem. Soc. Perkin Trans. 2*, pp. 487–492.

[bb35] Takahashi, M., Sekine, N., Fujita, T., Watanabe, S., Yamaguchi, K. & Sakamoto, M. (1998*a*). *J. Am. Chem. Soc.* **120**, 12770–12776.

