# Supplementary material for: Structures of S-(pyridin-2-yl) 4-nitro­benzo­thio­ate, S-(pyridin-2-yl) 4-methyl­benzo­thio­ate and S-(pyridin-2-yl) 4-meth­oxy­benzo­thio­ate: building blocks for low-symmetry multifunctional tetra­pyrroles
Source: Acta Crystallogr E Crystallogr Commun. 2023 Feb 9;79(Pt 3):157–62. doi: 10.1107/S2056989023001056 (PMC9993908; doi:10.1107/S2056989023001056)
Supplement: Supplementary file 8 [file e-79-00157-sup8.pdf]

Structures of *S*-(pyridin-2-yl) 4-nitrobenzothioate, *S*-(pyridin-2-yl) 4-methylbenzothioate, and *S*- (pyridin-2-yl) 4-methoxybenzothioate – building blocks for low symmetry, multifunctional tetrapyrroles.

Harry C. Sample,[a]* Brendan Twamley[b] and Mathias O. Senge[a]

[a]School of Chemistry, Chair of Organic Chemistry, Trinity Biomedical Sciences Institute, Trinity College Dublin, 152 - 160 Pearse St., D02 R590, Dublin, Ireland, and [b]School of Chemistry, Trinity College Dublin, College Green, Dublin 2, Ireland

Supporting Information

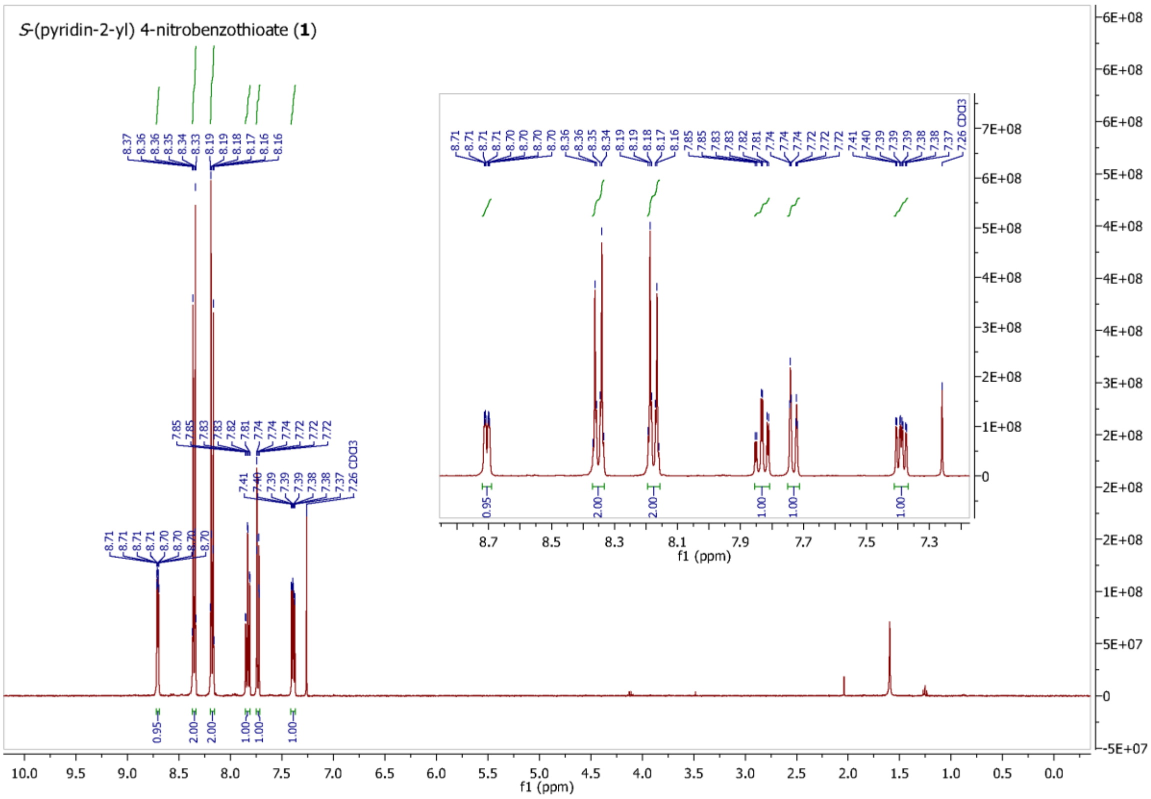

Fig. S1. ^{1}H NMR spectra of *S*-(pyridin-2-yl)-4-nitrobenzothioate (**1**) ($CDCl_3$, 400 MHz, 298 K). Chemical shifts were determined with respect to the residual solvent peak.

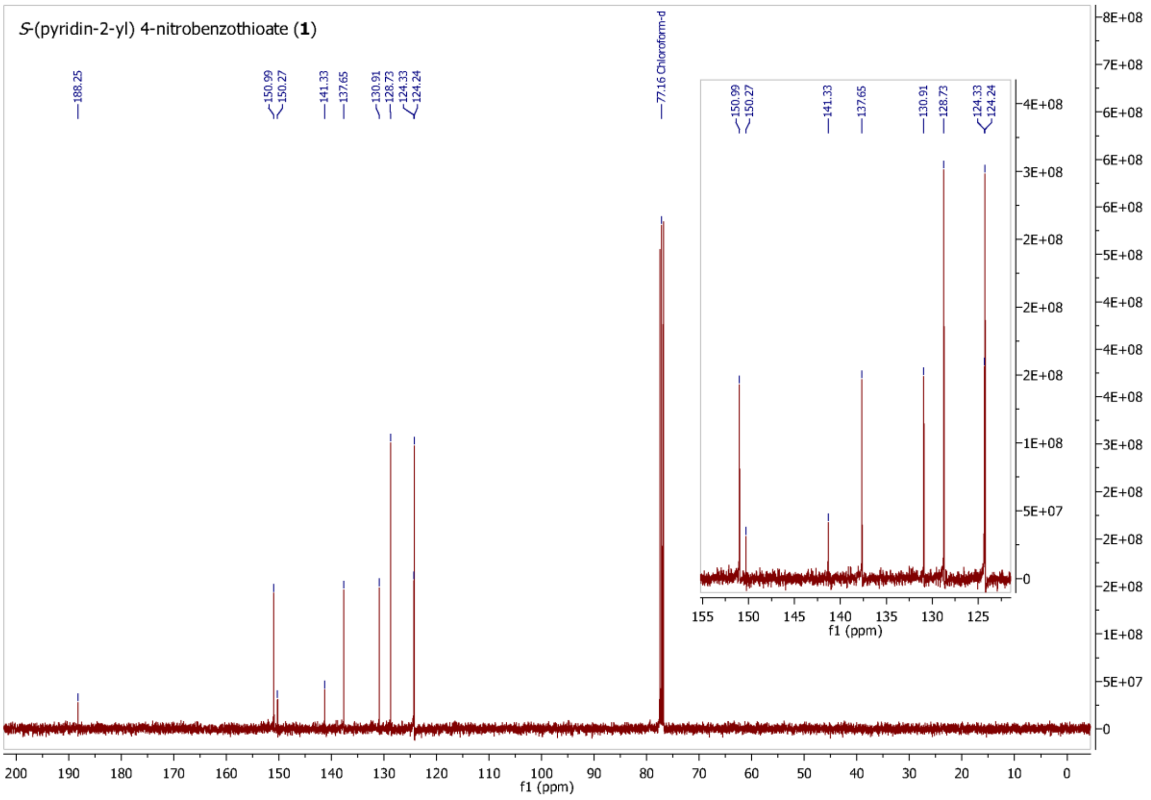

Fig. S2. ^{13}C NMR spectra of *S*-(pyridin-2-yl)-4-nitrobenzothioate (**1**) ($CDCl_3$, 101 MHz, 298 K). Chemical shifts were determined with respect to the residual solvent peak.

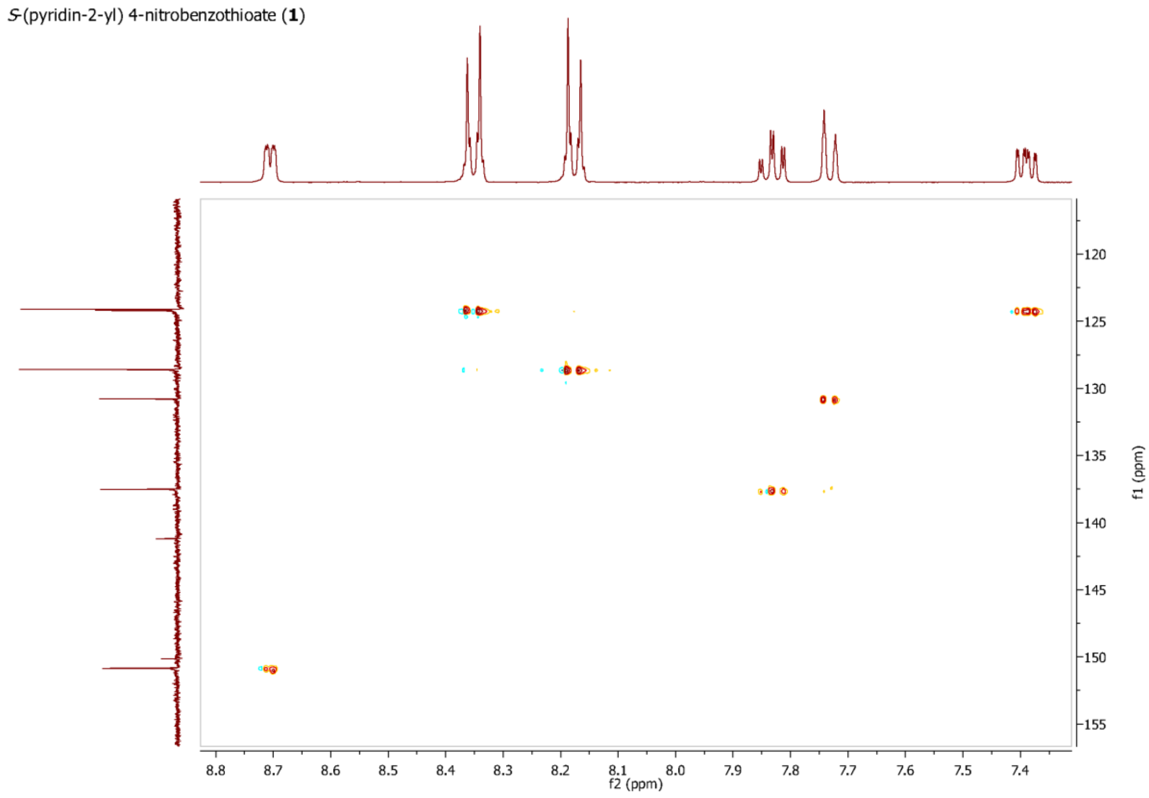

Fig. S3. ^{1}H-^{13}C HSQC spectra of *S*-(pyridin-2-yl)-4-nitrobenzothioate (**1**) ($CDCl_3$, 298 K). Spectra were aligned using data from the respective ^{1}H and ^{13}C NMR spectra.

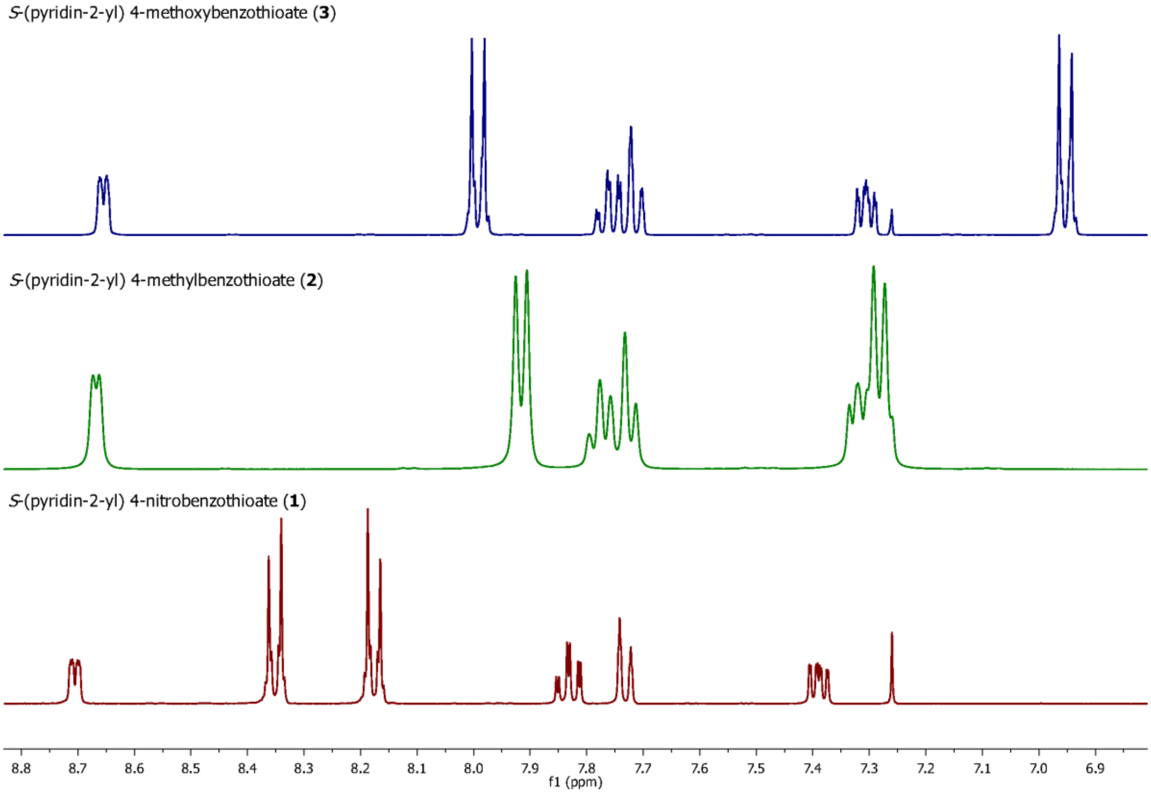

Fig. S4. Stacked ^{1}H NMR spectra of compounds **1**, **2**, and **3**. Spectra were aligned using the residual solvent peak.
